# Empirical validation of ProteinMPNN’s efficiency in enhancing protein fitness

**DOI:** 10.3389/fgene.2023.1347667

**Published:** 2024-01-11

**Authors:** Tianshu Wang, Xiaocheng Jin, Xiaoli Lu, Xiaoping Min, Shengxiang Ge, Shaowei Li

**Affiliations:** ^1^ School of Informatics, Institute of Artificial Intelligence, Xiamen University, Xiamen, China; ^2^ State Key Laboratory of Vaccines for Infectious Diseases, Xiamen University, Xiamen, China; ^3^ National Institute of Diagnostics and Vaccine Development in Infectious Diseases, Xiamen University, Xiamen, China; ^4^ State Key Laboratory of Molecular Vaccinology and Molecular Diagnostics, Xiamen University, Xiamen, China; ^5^ School of Public Health, Xiamen University, Xiamen, China; ^6^ Information and Networking Center, Xiamen University, Xiamen, China

**Keywords:** protein engineering, mutation, ProteinMPNN, structural constraints, sequence design

## Abstract

**Introduction:** Protein engineering, which aims to improve the properties and functions of proteins, holds great research significance and application value. However, current models that predict the effects of amino acid substitutions often perform poorly when evaluated for precision. Recent research has shown that ProteinMPNN, a large-scale pre-training sequence design model based on protein structure, performs exceptionally well. It is capable of designing mutants with structures similar to the original protein. When applied to the field of protein engineering, the diverse designs for mutation positions generated by this model can be viewed as a more precise mutation range.

**Methods:** We collected three biological experimental datasets and compared the design results of ProteinMPNN for wild-type proteins with the experimental datasets to verify the ability of ProteinMPNN in improving protein fitness.

**Results:** The validation on biological experimental datasets shows that ProteinMPNN has the ability to design mutation types with higher fitness in single and multi-point mutations. We have verified the high accuracy of ProteinMPNN in protein engineering tasks from both positive and negative perspectives.

**Discussion:** Our research indicates that using large-scale pre trained models to design protein mutants provides a new approach for protein engineering, providing strong support for guiding biological experiments and applications in biotechnology.

## 1 Introduction

Protein engineering holds considerable importance in biotechnology and biomedicine, which embodies the modification of natural sequences found in nature to optimize the properties and functions of proteins, especially enzymes and it has been widely applied in many fields (Narayanan et al., 2021). However, the experimental measurement of protein properties and functions is laborious and is applicable only to proteins that can be purified (Stevens, 2000). Currently, the prevalent methods are rational design In Silicon and directed evolution screened by computational methods.

In computational protein engineering, the most common approach is to combine machine learning methods, statistical potentials, physical and chemical properties, sequence features, and evolutionary information to construct models or energy functions to predict the mutation effects of protein sequences after amino acid substitution. Certain endeavors have made progress in specific downstream tasks, such as predicting enzyme catalytic activity (Li et al., 2021), solubility (Khurana et al., 2018; Chen et al., 2021) and stability of natural proteins (Broom et al., 2020; Chen et al., 2020; Li et al., 2020; Benevenuta et al., 2021; Wang et al., 2023). Particularly noteworthy is the work predicting the thermal stability of proteins based on the difference in folding free energy before and after mutation.

The performances of these methods have been evaluated and compared across different datasets of experimentally characterized mutants, demonstrating that while all methods reflect correct trends in their predictions, most are overly optimistic about their actual performance (Benevenuta et al., 2023). When applied to independent test sets, some models that claimed to achieve 70%–80% accuracy at the time of publication, only achieved an actual accuracy rate of 20% (Broom et al., 2020; Pucci et al., 2022). Such results fail to meet the requirements for guiding wet experiments and limit the further development of protein engineering in directional evolution strategies.

Deep learning model AlphaFold2 has made a significant breakthrough in predicting protein structure from amino acid sequences (Jumper et al., 2021), addressing a fundamental problem in protein biochemistry. The corresponding inverse problem, which predicts sequences and sequence mutations that can fold into the same or similar structures from the protein structure, has also made significant progress (Ferruz et al., 2022; Hesslow et al., 2022; Madani et al., 2023). ProteinMPNN, a deep learning method for protein sequence design, has shown outstanding performance in computational and experimental tests (Dauparas et al., 2022). Working on the backbone of natural proteins, ProteinMPNN achieves a sequence recovery rate of 52.4%. Nevertheless, the diverse design results for sites that are not perfectly replicated in the sequence may also possess significant research value. A recent study indicates that high-confidence erroneous predictions on the wild-type could potentially identify mutation locations and possible targets for protein engineering (Kulikova et al., 2021). Sequences designed by ProteinMPNN, which fold similarly to the wild type, can be viewed as mutants of the wild type protein. ProteinMPNN obtains diverse design results through the deviation of natural protein sequence recovery results, and defines a concise and accurate mutation space. This process potentially results in proteins with improved functions and properties.

In this study, the diverse results designed at each site in the sequence are considered to be the mutation space under structural constraints for that position. We tested this method using a biological experimental dataset. Our comparative analysis of both single and multiple point mutations datasets showed that the sequences, designed by ProteinMPNN using natural proteins as templates, demonstrated considerable effectiveness. Especially in the design for single point, up to 60.8% of the results within the defined mutation space were proved to be effective by the experimental dataset, far superior to methods and tools for predicting the effect of point mutations. This suggests that ProteinMPNN has the capability to design mutants with improved adaptability, which carries significant implications for determining a plausible mutation space, reducing harmful mutations, expediting targeted mutagenesis, and steering biological experiments.

## 2 Methods

### 2.1 TEM-1 *β*-lactamase

TEM-1 *β*-lactamase protein has been widely studied for its resistance to penicillin antibiotics in *E. coli*, and its mutation effects have also attracted much attention (Jacquier et al., 2013). Firnberg et al. (2014) conducted comprehensive research on gene point mutations, codon mutations, and nearly all possible single amino acid substitutions of TEM-1 *β*-lactamase, proposed a comprehensive distribution of fitness effects (DFE) and established a fitness landscape model for the evolution of TEM-1 protein. The fitness scores of 95.6% (5212/5453) of the mutants in the TEM-1 sequence mutation space were experimentally measured. In this task, except for the signal sequence composed of the first 25 residues, ProteinMPNN will sequentially design the remaining positions in the sequence and compare the design outcomes with experimental data to determine the proportion of mutants in the designed results that can maintain or enhance the fitness of TEM-1.

ProteinMPNN claims the ability to make inferences at higher temperatures. In some deep learning models, appropriately using the temperature parameter can fine-tune the model’s predictive probability distribution to be either smoother or sharper. During the design process of ProteinMPNN, the temperature sampling parameter adjusts the probability values for the 20 amino acids at each position in the sequence, thereby controlling the diversity of the design outcomes. The temperature sampling parameter of ProteinMPNN ranges from 0 to 1, with higher values leading to increased diversity in the designed results. Diversity is crucial for this task, low diversity may lead to the loss of many beneficial mutations, while diversity greater than 0.5 can result in a noticeable decrease in sequence recovery rates. This might cause ProteinMPNN to define excessively large mutation ranges for each position, rendering it less meaningful. Therefore, this task is conducted at three temperature samples: 0.1, 0.3, and 0.5.

Some hypotheses suggest that enzyme thermostability can affect protein abundance, thus influencing protein catalytic activity (fitness) (DePristo et al., 2005; Camps et al., 2007; Tokuriki et al., 2007; Wylie and Shakhnovich, 2011). The authors predicted ΔΔ*G* (Δ*G*_wild-type_ − Δ*G*_mutant_) values for 4,783 missense mutations of TEM-1 using tools like PoPMusic (Dehouck et al., 2011). They combined the observed melting temperature and fitness correlation from experiments, demonstrating a relationship between the thermostability and fitness of individual proteins. In a related work on model-guided protein sequence design, landscape training data was also obtained from this dataset, and the fitness was considered as thermodynamic stability (Ren et al., 2022).

Currently, there are many tools for predicting the stability changes after single point mutations in protein sequences. We selected three representative methods. PoPMusic, based on folding free energy calculation, and mCSM(Pires et al., 2014), which using graph-based signatures, are well-tested prediction tools, with superior performance on most datasets compared to other methods. DeepDDG (Cao et al., 2019), which also performs well on some independent datasets, is considered a representative of stability prediction using deep learning algorithm. While ProteinMPNN is not a predictor, its application in single-point design results in a proportion of truly beneficial outcomes within its design range (mutation space). This is conceptually similar to precision in prediction tools, as this metric largely determines the success rate of biological experiments guided by their respective results. Hence, we will use these three methods to predict the saturation mutations of all sites on TEM-1, and compare their precision with the results of ProteinMPNN.

### 2.2 Green fluorescent protein (GFP)

Green fluorescent protein (GFP) is a protein originally derived from the Victoria multibarrelled luminous jellyfish. It can be excited by light ranging from blue to ultraviolet and emits green fluorescence. The structure of GFP is defined by a sequence of 238 amino acid residues, forming a tightly packed structure. This structure includes 11 *β*-pleated sheets arranged in a barrel shape, which surrounds the central chromophore, S65-Y66-G67 (Ormö et al., 1996). In molecular biology, medicine, and cell biology, GFP is commonly used as a biomarker due to its stability and the fact that its chromophore is formed through self-catalyzed cyclization without the need for cofactors. Moreover, its stable structural characteristics and reliable functional performance have made it an ideal subject for exploring the complex relationships between protein sequences, structures, and functions, as well as for establishing comprehensive landscapes of protein fitness in recent years. Sarkisyan et al. (2016) have contributed remarkable work to the community. They experimentally measured the fluorescence level of more than 50,000 sequences synthesized through random mutagenesis in mutants. These mutants were obtained by making single or multiple residue substitutions in the wild-type GFP(avGFP) sequence from position 3 to 237. In the original work, the authors reported that more than 75% of mutant fluorescence intensity was lower than that of the wild type. But the reduced fluorescence was more likely to result from the combined effect of multiple residue mutations or the accumulation of harmful mutations. Single mutations have a small effect on fluorescence intensity, but 9.4% of single mutations still result in a more than 5-fold decrease in fluorescence. A related work collated the fitness landscape data of this protein, providing fluorescence scores of 1051 single residue mutants, of which 953 were considered to be light (on) and 98 were dark (off) (Masso, 2020).

Considering the fact that most single point mutations have limited effects on fluorescence function, ProteinMPNN designed single-point mutant sequence still has a high probability of maintaining fluorescence. However, the current experimental data on single-residue mutants, in comparison to the entire sequence’s mutational space, remains sparse. The results generated by ProteinMPNN cannot be fully compared with the experimental data. Therefore, in this task, we will use the GFP’s off mutants as the comparison object of the ProteinMPNN’s design results. Within the single residue mutation dataset, a total of 98 mutant variants are distributed across 66 distinct sites, indicating that mutations occurring within the mutation space of these sites can significantly diminish fluorescence intensity. Our aim is to utilize ProteinMPNN to design these 66 positions. We intend to assess whether ProteinMPNN can, in regions with uncertain mutational effects, avoid deleterious mutations. ProteinMPNN needs to delineate a small and reasonable mutation space for these 66 positions, and this mutation space should have minimal overlap with the off mutants in the dataset. The criterion is that the probability of harmful mutations in the ProteinMPNN design results, denoted as *P*, should be lower than that of random mutations, *P*_rand_. i.e.,

$$P=\frac{N}{D_{\text{MPNN}}}$$ (1)

$$P_{\text{rand}}=\frac{D_{\text{off}}}{D}$$ (2)

the mutation space of all 66 positions involved in the mutation is *D*, with a size of 66 × 19 = 1254. The set of 98 harmful mutations in the dataset is *D*_off_, the mutation space defined by ProteinMPNN is *D*_MPNN_, where the harmful mutations *N* are the intersection of *D*_off_ and *D*_MPNN_. In this task, the design is still carried out under three temperature samples of 0.1, 0.3, and 0.5.

### 2.3 PTMUL

The mutational space and complexity of multiple point mutations are far greater than single point mutations, and the impact on protein structure changes and properties and functions is also far more significant than single point mutations. Introducing multiple amino acid substitutions at multiple positions in site-directed mutagenes is also a common method. However, most of the current work in downstream tasks only predicts the effect of single point mutations, and there are few neural network models that predict the effect of multiple point mutations. The primary reason for this might be the severe lack of reliable data, restricting neural networks from capturing multi-point mutational synergies. In this case, a method for designing protein mutation sequences based on structural design may be a solution to this problem. The PTMUL dataset is a thermodynamic dataset containing only multiple point mutations (Montanucci et al., 2019), including 914 records of multiple point mutations from 91 protein structures and 77 clusters. Each mutant type includes mutations with a number of mutation sites ranging from 2 to 10. This dataset is sourced from Protherm (Kumar et al., 2006), which is one of the most extensively used resources in protein stability research. We obtained structural information for the relevant proteins from the PDB website. After excluding proteins with excessively short sequences, incomplete structures, and those lacking favorable mutation records, we ultimately collected 251 mutation records from 49 protein variants, comprising 185 different mutation site combinations.

In this task, ProteinMPNN is configured with a temperature sample of 0.3. For each mutation type involving three or fewer sites, 50 results are designed. For mutation types with more than three sites, 100 results are generated. While this approach may result in duplicate sequences within the outcomes, the results obtained after manually excluding these duplicates ensure that ProteinMPNN has conducted thorough designs for each mutation combination. As an illustration, for *E. coli* ribonuclease H (PDB: 2RN2), there are a total of 18 records in the PTMUL dataset. Among these, 6 records indicate a decrease in stability, while the remaining 12 records show an increase in stability. Within these 12 records showing increased stability, there are seven different combinations of mutation sites. ProteinMPNN is used to conduct comprehensive designs for each of these seven multi-point mutation combinations. The obtained mutation results are then compared with the mutation records of the 2RN2 protein from the dataset, verifying whether ProteinMPNN retains the ability to design mutations with higher fitness for the complex task of multi-point protein mutations.

## 3 Results

### 3.1 TEM-1 *β*-lactamase

The fitness dataset for TEM-1 *β*-lactamase protein is a saturated dataset, enabling precise calculations of both ProteinMPNN’s accuracy and third-party prediction tool precision in this task. In this dataset, we have defined mutations with a Fitness score above 1.0 as those capable of maintaining or improving the fitness of TEM-1. The proportion of such mutations in the dataset is 21.7% (1094/5032, excluding signal sequences). Consequently, in the results designed by ProteinMPNN, the proportion of mutant types that can maintain or improve adaptability should at least surpass this benchmark to indicate that the method is effective. For third-party prediction tools, their precision must also be at least higher than this proportion to prove that the prediction model shows the correct trends. The purpose of using different temperature samples in this task is to control the diversity of design results. Although a lower temperature sample may increase the proportion of advantageous mutations in the results, it can only recover some sites to the same unique residues as the wild-type, reducing the diversity of design results and missing out on some mutant results that may improve fitness. Raising the temperature sample results in more comprehensive coverage of advantageous mutations, but it may lead to an overall decrease in precision. Our statistical results align with these expectations (Table 1).

**TABLE 1:** The results of TEM-1 designed by ProteinMPNN.

Temperature sample	Sites with missense mutation	All results	Maintain of improve	Ratio (%)
0.1	125	171	104	60.8
0.3	155	296	162	54.7
0.5	186	480	244	50.8

In the context of the TEM-1 sequence design task, a total of 264 positions were individually designed. When the temperature sample was set at 0.1, 139 positions recovered to the same amino acids as the wild-type sequence, while the remaining 125 positions yielded 171 mutation results. Among these, 104 were empirically validated as mutations that maintained or improved fitness, constituting 60.8% (104/171). With the temperature sample increased to 0.3 and 0.5, the figures became 162/296 and 244/480, with proportions of 54.7% and 50.8%, respectively.

**FIGURE 1:**
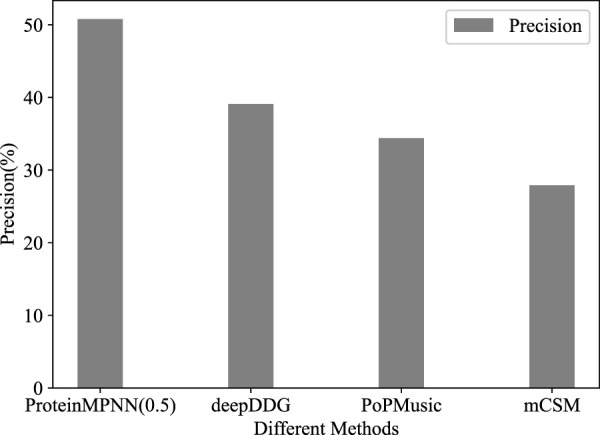
The precision of different methods in TEM-1 task.

Using third-party tools, out of 573 stability mutations predicted by mCSM, 160 were experimentally confirmed as stable. The results from PoPMusic and deepDDG were 206/600 and 518/203, with precisions of 27.9%, 34.3%, and 39.1%, respectively (Figure 1). This indicates that ProteinMPNN significantly outperforms methods for predicting the effects of amino acid substitutions in this task.

**FIGURE 2:**
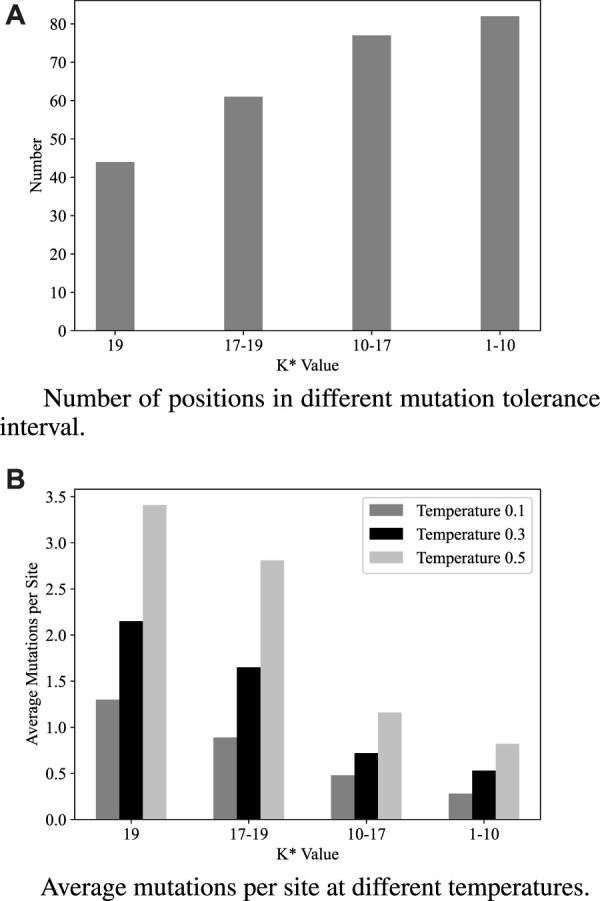
ProteinMPNN designed at different K* value. **(A)** The figure shows the Number of positions in different K* value interval, and the K * value indicates the tolerance of the site to mutations. **(B)** At three different temperatures, ProteinMPNN had a higher average design number for sites with stronger mutation tolerance.

The tolerance of each position on the TEM-1 sequence towards mutations was expressed using K* values in the work by Firnberg et al. (2014). A K* value of 20 indicates that all 19 amino acid substitutions result in fitness equivalent to the wild-type amino acid, while a K* value of 1 corresponds to all mutations causing complete loss of activity at that sequence position (Figure 2A). Combining the K * value with the design results of ProteinMPNN for analysis: There were a total of 44 sites with K * values greater than 19. ProteinMPNN designed 150 mutant types for these 44 sites at a temperature of 0.5, with an average of 3.4 results designed for each site. There were 61 positions with K* values in the range of 17–19, for which a total of 172 mutations were designed, averaging 2.81 mutations per position. For positions with K* values ranging from 10–17 and below 10, there were 77 and 82 positions, respectively, and ProteinMPNN designed 90 and 68 mutations for these groups, as depicted in Figure 2. From the figure, it can be observed that ProteinMPNN introduced the most diverse designs for the 44 positions with K* values greater than 19. These positions had the fewest numbers, but the highest average design results per position (Figure 2B). For positions with lower tolerance (K* values less than 10), ProteinMPNN had a higher probability of directly reverting them to the same amino acids as the wild-type sequence. These results can be interpreted as ProteinMPNN ‘focusing’ on positions with high tolerance, where design feasibility is greater. This suggests that, without any external intervention, ProteinMPNN can autonomously identify positions in the sequence with higher design potential and thoroughly engineer them. This ability is one of the reasons for ProteinMPNN’s outstanding performance in this task.

### 3.2 Green fluorescent protein (GFP)

ProteinMPNN’s design results for the 66 positions in the GFP sequence also increase with the rising temperature. Table 2 shows the number of harmful mutations (Off), the total number of mutations, and the ratio in the design results.

**TABLE 2:** The results of GFP designed by ProteinMPNN.

Temperature sample	Sites with off mutation	All results	Maintain of improve	Ratio (%)
0.1	1	123	1	0.8
0.3	4	166	5	3.0
0.5	5	217	6	2.7

From the specific design results, ProteinMPNN produced 123 results for these 66 positions at a temperature of 0.1. Only one mutant type (Q181L) of 98 harmful mutations was designed at site 181. At temperatures of 0.3 and 0.5, the number of sites designed as harmful mutations was only 4 and 5. It is noteworthy that previous studies have shown that glycine on the luminescent residue group is the most important functional site, and any substitution of this site with any amino acid will result in complete loss of fluorescence. ProteinMPNN, however, perfectly restored the wild-type glycine at this position at temperatures of 0.1, 0.3, and 0.5, providing the foundation for improving or maintaining fluorescence levels in the mutant sequences.

When the temperature increases from 0.1 to 0.3 or 0.5, the diversity of the design results increases, and the proportion of harmful mutations also increases, indicating a higher proportion of improving or maintaining the fitness of wild-type proteins in the design results under smaller temperature parameters. The proportion of harmful mutations in the entire mutation space is *P*_rand_ = 7.8%. Moreover, at all three temperatures, the probability of harmful mutations in ProteinMPNN’s design results (referred to as *P*) is consistently lower than *P*_rand_, indicating that ProteinMPNN defines mutation spaces for the designed positions with very minimal overlap with harmful mutations in the dataset. This proves that ProteinMPNN still performs well in GFP fitness (fluorescence intensity) tasks. Combined with the results from the TEM-1 task, ProteinMPNN can adequately design high-tolerance positions. When it comes to designing positions with potentially harmful mutational effects (e.g., the 66 sites in GFP), ProteinMPNN can still avoid the majority of harmful mutations and ensure their properties and functionality.

### 3.3 PTMUL

PTMUL is not a saturated mutation dataset. Among 181 position combinations, there is an average of only 1.4 data records for each combination. Consequently, determining whether ProteinMPNN’s design results accurately hit the target is exceedingly challenging in this context. Due to this dataset’s characteristics, it is not feasible to calculate the proportion of ProteinMPNN design results that genuinely improve protein stability in this task. In this scenario, we use the concept of a hitting rate for result evaluation. For example, considering 2RN2, mutations like K91G:K95G and D94R:K95G can be directly designed by ProteinMPNN, meaning these mutations are directly hit in the design results and are considered direct hits. In contrast, for the A52:V74 mutation site combination, mutations A52V and V74L appeared separately in the design results but were not combined in a single sequence. This situation is referred to as an indirect hit. The results of this task indicate that among a total of 49 proteins, ProteinMPNN directly designed 12 mutations that improved stability. Out of the 185 mutation combinations, 24 were directly hit by ProteinMPNN, while 38 were indirectly hit. In total, 269 different positions were involved in the design, and 126 of them had more stable amino acid substitutions found by ProteinMPNN, accounting for 46.8%. These findings demonstrate ProteinMPNN’s capability in designing mutations with higher fitness and accurately defining mutation spaces in multi-site tasks.

## 4 Discussion

Modeling In Silicon aim to guide wet lab experiments based on computational results, ultimately translating into real-world applications in engineering. Therefore, evaluating the model’s computational results with experimental biological data holds significant importance. The current popular prediction models have serious data scarcity issues in the datasets used in specific downstream tasks, which limits the generalization ability of prediction models. Therefore, few methods can consistently perform well in different test sets (Pucci et al., 2022; Benevenuta et al., 2023). Moreover, since most mutations lead to decreased fitness, datasets are dominated by harmful mutations, causing prediction models to overfit on predicting harmful mutations (Montanucci et al., 2019; Benevenuta et al., 2023; Diaz et al., 2023). Presently, prediction models are primarily evaluated using Pearson correlation coefficients, classification accuracy, and error, with high accuracy often stemming from the prediction of the relatively high proportion of harmful mutations in the test set (Diaz et al., 2023). However, precision is the real measure of a model’s importance. What people are more concerned about is the true proportion of beneficial mutations in the prediction results, as this directly affects the success rate of lab experiments.

Despite some previous efforts to address these issues, including extending relevant datasets and achieving certain results (Diaz et al., 2023), the severe lack of real data obtained from biological experiments continues to plague the community. In this context, the utilization of AI models with large-scale pretraining techniques could be one effective solution to this problem. ProteinMPNN is a model trained extensively on structural data, and it can generate sequences that, while recovering the main chain structure, can reliably and accurately fold into a natural protein scaffold. Introducing this model into the field of protein engineering allows us to harness the rich knowledge it has acquired from massive datasets to directly design sequences with improved properties and functions. This approach helps mitigate the severe lack of high-quality data in downstream task datasets. Some experiments have already successfully used ProteinMPNN’s design results to guide the optimization of protein properties and functions (Sumida et al., 2023).

As we look toward the future of protein engineering, large-scale pretrained models, based on either structural or sequence data, are poised to revolutionize the field. These advanced computational tools are not merely incremental improvements but represent a paradigm shift in how we approach the design and optimization of proteins. These models offer the unique advantage of tapping into the vast amount of sequence and structural data accumulated over years of research. By leveraging this wealth of information, pretrained models can uncover patterns and relationships not readily apparent to human researchers, leading to novel insights and the discovery of unprecedented protein functionalities. Moreover, there is immense potential for these models to become more sophisticated through continuous learning. As they encounter new data from ongoing protein engineering experiments, the models can enhance their capabilities, becoming ever more accurate and reliable. This capacity for self-improvement will ensure that the models remain at the cutting-edge of technology, dynamically evolving alongside scientific progress. We anticipate more widespread applications of large-scale pretrained models, whether based on structure or sequence, in the protein engineering field. These models hold the promise of bringing significant advancements, providing powerful tools for more effectively improving protein properties and functions. This will contribute to driving more scientific breakthroughs in the field of protein.

## 5 Conclusion

In this study, we propose a method that involves using protein sequence design tools to create mutants capable of folding into the same or similar structures while preserving the original structure. We applied the next-generation sequence design tool, ProteinMPNN, to design proteins from the tem-1, GFP, and PTMUL datasets and validated the approach using experimental data. Our results demonstrate that ProteinMPNN’s diverse restoration of wild-type sequence residues yields a more accurate mutation space, including a considerable proportion of mutants with improved fitness. Given the current limitations in the accuracy of mutation impact prediction tools, this method potentially offers a more reliable choice for biological experiments. Despite the limitations of protein mutation datasets, especially the lack of saturation, which hinders further progress in our work, the outstanding performance of ProteinMPNN has already proven this approach to be a new solution for the field of protein engineering. As large-scale pre-training models continue to advance and protein research deepens, we have every reason to believe that ProteinMPNN and similar methods will continue to play a crucial role in providing stronger support for protein engineering and the field of biomedicine in the future.

## Data Availability

Publicly available datasets were analyzed in this study. This data can be found here: https://academic.oup.com/mbe/article/31/6/1581/2925654, https://figshare.com/articles/dataset/Local_fitness_landscape_of_the_green_fluorescent_protein/3102154 https://github.com/protddg-bench/protddg-bench.
